# Two Cases of Dengue Fever Imported from Egypt to Russia, 2017

**DOI:** 10.3201/eid2404.172131

**Published:** 2018-04

**Authors:** Muhammad A. Saifullin, Victor P. Laritchev, Yana E. Grigorieva, Nadezhda N. Zvereva, Anna M. Domkina, Ruslan F. Saifullin, Marina V. Bazarova, Yulia A. Akinshina, Ludmila S. Karan, Aleksandr M. Butenko

**Affiliations:** Research Institute of Epidemiology and Microbiology n.a. N.F. Gamalei, Moscow, Russian Federation (M.A. Saifullin, V.P. Laritchev, Y.A. Akinshina, A.M. Butenko);; Municipal Infectious Diseases Hospital No. 1, Moscow (M.A. Saifullin, R.F. Saifullin, M.V. Bazarova);; Central Scientific Research Institute of Epidemiology, Moscow (Y.E. Grigorieva, L.S. Karan);; Pirogov Russian National Research Medical University, Moscow (N.N. Zvereva, R.F. Saifullin);; Municipal Infectious Diseases Hospital No. 2, Moscow (A.M. Domkina)

**Keywords:** fever, dengue, Egypt, emergence, viruses, Russia, dengue virus

## Abstract

In 2017, two cases of dengue fever were imported from Hurghada, Egypt, where dengue fever was not considered endemic, to Moscow. These cases show how emergence of dengue fever in popular resort regions on the coast of the Red Sea can spread infection to countries where it is not endemic.

From 2012, when registration of dengue fever cases became official in the Russian Federation, through December 2017, ≈700 cases of imported dengue fever have been registered in Russia (*1*). Of these, ≈90% of cases were associated with travel to southern and Southeast Asia, mainly Thailand. In 2015, almost 2.5 million Russian citizens visited Egypt (the fifth most popular destination for tourists from Russia) (*2*). After a plane crash caused by a terrorist attack on October 31, 2015, air connections and tours to Egypt were stopped; however, many Russian citizens with relatives or real estate in resort areas of Egypt still travel there.

In Africa, dengue fever is endemic to 34 countries, including Egypt. During 1960–2010, a total of 20 confirmed outbreaks in 15 countries were registered (*3*). For the past 7 years, outbreaks in East Africa have been registered in Somalia (2011, 2013), Kenya (2011, 2013, 2014), Tanzania (2013, 2014), and Mozambique (2014). In 2017, outbreaks were registered in 8 countries, 6 in West Africa and 2 in East Africa (Kenya, Seychelles). In Sudan, which is bordered on the north by Egypt, outbreaks were registered in 2010, 2011, and 2014 (*4**,**5*).

Dengue fever cases have been registered earlier in Egypt; the last outbreak was in 2015 in Dairut, but there are no data about outbreaks in cities on the coast of the Red Sea (e.g., Hurghada, Sharm El-Sheikh, and Dahab), which have become resort destinations for Russian citizens (*6*). In October 2017, the health department of the Red Sea governorate reported cases of dengue fever in El Qoseir, a city 145 km south of Hurghada, with a population of ≈50,000. Preliminary results indicated that 1,200–2,500 persons were infected (*7**,**8*). Since November 2017, six cases of dengue among tourists returning from Hurghada (*9**,**10*) were reported in Belgium (*1*), Austria (*1*), and Germany (*4*). Until 2017, to our knowledge, no cases of dengue fever had been imported from Egypt to Russia. We report 2 cases in persons returning to Russia after visiting Hurghada.

Patient 1 was a 63-year-old female Moscow resident. During October 12–28, 2017, she had visited relatives in Hurghada, stayed in their apartment, and noted multiple insect bites. On October 18, she experienced acute onset of chills, fever, and aches. She did not seek medical care and did not measure her temperature. She took acetaminophen to control her symptoms. Over 10 days, her health gradually improved, but the fatigue remained. After returning to Moscow, she noted low-grade fever (up to 37.0°С) and was hospitalized in Infectious Clinical Hospital No. 1 with a diagnosis of fever of unknown origin. At admission, she complained of fatigue, restless sleep, dizziness, and tinnitus. Her general condition was stable and her temperature was 37.5°С; physical examination revealed no significant abnormalities. Complete blood count, urinalysis, and biochemical test results were within normal limits. On day 32 after symptom onset, serum testing by IgM antibody capture (MAC)–ELISA (in-house kit) detected IgM (titer 1:1,600) and IgG ELISA detected IgG (titer 1:12,800) against dengue virus (DENV). On day 34, real-time PCR (Dengue Real-TM Genotype; Sacace Biotechnologies, Como, Italy), used according to the manufacturer’s instructions, detected DENV type 2 RNA in urine (detected on 35th amplification cycle). Patient 1 received symptomatic treatment and was discharged after improvement with no complications.

Patient 2 was a 49-year-old male Moscow resident. During October 28–November 17, 2017, he had vacationed in Hurghada, stayed in a hotel, and noted insect bites. He denied having had contact with ill persons. On November 15, he experienced acute onset of chills, fatigue, and fever (39.0°С). He did not seek medical care and did not measure his temperature again. He took nonsteroidal antiinflammatory medications to control his symptoms. After returning to Moscow (via Istanbul), he was taken by emergency ambulance to Municipal Infectious Diseases Hospital No. 2, where he was hospitalized for suspected malaria. At admission, he had fever and rash. His general condition was stable and his temperature was 39.0°С. Physical examination revealed mild macular rash and liver enlargement 3 cm below the edge of the costal arch. Complete blood count indicated thrombocytopenia (106 × 10^9^ thrombocytes/L) and leukopenia (1.8 × 10^9^ cells/L); urinalysis and biochemical test results were within normal limits. On day 7 after symptom onset, real-time PCR (Dengue Real-TM Genotype) detected DENV type 2 RNA in blood (25th cycle) and urine (30th cycle). Patient 2 received symptomatic treatment and was discharged after improvement with no complications.

The emergence of dengue fever on the coast of the Red Sea, a popular resort region for tourists from many countries, can lead to increased importation to countries where it is not endemic. Considering these 2 cases of dengue fever imported from Hurghada, Egypt, tourists should be informed about the risk for DENV infection before they travel, preventive measures should be explained, and tourists should be advised to seek medical care early if they experience symptoms. 
